# A new simple method for quantification and locating P and N reserves in microalgal cells based on energy-filtered transmission electron microscopy (EFTEM) elemental maps

**DOI:** 10.1371/journal.pone.0208830

**Published:** 2018-12-11

**Authors:** Tatiana Ismagulova, Anastasia Shebanova, Olga Gorelova, Olga Baulina, Alexei Solovchenko

**Affiliations:** 1 Department of Bioengineering, Faculty of Biology, Lomonosov Moscow State University, Moscow, Russia; 2 Eurasian Centre for Food Security, Lomonosov Moscow State University, Moscow, Russia; Universidade Federal de Juiz de Fora, BRAZIL

## Abstract

We established a new simple approach to study phosphorus (P) and nitrogen (N) reserves at subcellular level potentially applicable to various types of cells capable of accumulating P- and/or N-rich inclusions. Here, we report on using this approach for locating and assessing the abundance of the P and N reserves in microalgal and cyanobacterial cells. The approach includes separation of the signal from P- or N-rich structures from noise on the energy-filtered transmission electron microscopy (EFTEM) P- or N-maps. The separation includes (i) relative entropy estimation for each pixel of the map, (ii) binary thresholding of the map, and (iii) segmenting the image to assess the inclusion relative area and localization in the cell section. The separation is based on comparing the *a posteriori* probability that a pixel of the map contains information about the sample vs. Gaussian *a priori* probability that the pixel contains noise. The difference is expressed as relative entropy value for the pixel; positive values are characteristic of the pixels containing the payload information about the sample. This is the first known method for quantification and locating at a subcellular level P-rich and N-rich inclusions including tiny (< 180 nm) structures. We demonstrated the applicability of the proposed method both to the cells of eukaryotic green microalgae and cyanobacteria. Using the new method, we elucidated the heterogeneity of the studied cells in accumulation of P and N reserves across different species. The proposed approach will be handy for any cytological and microbiological study requiring a comparative assessment of subcellular distribution of cyanophycin, polyphosphates or other type of P- or N-rich inclusions. An added value is the potential of this approach for automation of the data processing and evaluation enabling an unprecedented increase of the EFTEM analysis throughput.

## Introduction

Microalgae are capable of accumulating phosphorus- and nitrogen-rich reserve compounds built from the nutrients sequestered from the environment. A considerable interest to these organisms and processes is fueled by the development of biotechnologies based on microalgal cultivation to respond to global environmental and socio-economic challenges related with the sustainable usage of the key nutrients. The promising approaches include the prevention of eutrophication by efficient bio-capturing of phosphorus (P) and nitrogen (N) by microalgae from urban and agricultural wastewater [1] with subsequent return of the recycled P and N to the field in form of microalgal biomass-based fertilizers [2,3]. Moreover, the dynamics of phytoplankton abundance and aquatic ecosystems productivity is tightly related with availability and distribution of both the nutrients, N and P [4–6]. The inorganic P taken up from the cultivation medium in the process known as ‘luxury uptake’ [7] is stored in form of phosphorus-rich inclusions (PRIs) harboring the cellular reserves of the nutrient P. Internal P reserves in all type of cells including cyanobacteria and eukaryote microalgae are constituted mainly by inorganic polyphosphate (PolyP) inclusions [8–10].

In a number of cyanobacterial and eukaryote microalgal species, the N taken up in excess of current metabolic requirement from the environment is deposited in the form of nitrogen-rich inclusions (NRIs). Cyanophycin (multi-L-arginyl-poly-[L-aspartic acid]) granules accommodate the NRIs in the majority of cyanobacteria [9]. In eukaryotes, the knowledge about the compounds of the NRIs is much more limited. Uric acid in the form of crystals in dinoflagellate microalgae [11–14] and guanine in the form of inclusions in a chlorophyte *Desmodesmus quadricauda* [15], a eustigmatophyte *Trachydiscus minutes* [15], and a dinophyte *Gonyaulax polyedra* [16–18] have been considered as candidate N-reserve compounds in the composition of the NRIs.

Understanding the mechanisms of the accumulation and expenditure of the intracellular macronutrient reserves is crucial for the control and prediction of the dynamics and productivity of natural populations and industrial cultures of single-celled phototrophs. Solving this problem presumes development of efficient methods for visualization and quantification of the PRIs and NRIs. One of the powerful analytical methods used to visualize the P and N reserves in cells at the subcellular scale is energy-filtered transmission electron microscopy (EFTEM) elemental mapping. An EFTEM N or P map reflects the distribution of N or P over the studied cell section. The EFTEM mapping is based on electron energy loss spectroscopy (EELS). It includes acquisition of so called post-edge images formed by detection of electrons that have lost energy corresponding to certain inner-shell ionization edge. The energy loss is characteristic for the element of interest. The resulting image is formed after subtraction of the background from the acquired post-edge image. During estimation of the background which depends on the chosen method (two-window, three-window, etc.) additional electron images called pre-edge images are recorded. The technique of recording and calculating elemental maps is described in more detail in standard textbooks [19,20]. Development of advanced software techniques combined with improved spectrometers, detectors, and electron optics made the elemental EFTEM mapping a valuable technique for fast and easy detecting light elements with a high sensitivity in biological samples [21–23].

The EFTEM mapping of light elements including the key macronutrients P and N was used in studies of plant-cyanobacterial symbiosis to visualize PRIs and cyanophycin granules in the cyanobiont cells [24,25]. Cyanophycin granules were also successfully mapped in recombinant strains of *Ralstonia eutropha* expressing the cyanophycin synthetase of *Anabaena* sp. [26]. EFTEM mapping was also successfully applied to eukaryotic microalgae for identification and localization of their PRIs and non-protein NRIs [27]. Cellular NRIs identified as uric acid were also visualized in symbiotic dinoflagellates by the elemental mapping [11,12].

Advantages of EFTEM mapping for detection of light elements such as P and N include a high sensitivity and the ability for visualization of PRIs and NRIs in cells. However, the literature available at the time of this research lacked reports on quantification PRIs and NRIs on EFTEM cell maps. Developing a quantitative approach based on this method is complicated by interferences in the EFTEM maps arising from the cellular structures unrelated to the inclusions of interest and noise. In this study, we proposed the use of a relative entropy measurement and binary thresholding of images to distinguish the signals from the inclusions of interest and other signals on the EFTEM maps. The proposed method facilitates evaluation of the area and localization of the inclusions in the cell. As far as we know, this is the first method for quantification of the cell PRIs and NRIs on EFTEM maps.

Thresholding is a well-known approach to image enhancement, segmentation and object detection used in a variety of image processing methods [28–32]. Among them, particularly interesting are techniques based on the concept of entropy. In the thresholding methods, two types of entropy are basically used: Shannon’s entropy [28,29,33] and relative entropy [34,35]. Detailed survey and comparative analysis of the entropy and relative entropy thresholding techniques were conducted by Chang et al. [36].

Analysis based on the relative entropy measurement used for building EFTEM maps from post-edge image and pre-edge images was first proposed by Trebbia et al. [37, 38]. They demonstrated that the relative entropy measurement can be valuable for building maps from noisy energy-filtered images. Novelty of the method developed in this work is constituted by (i) a simple separation of the payload signal from irrelevant signals and noise in the EFTEM map *via* binary thresholding using the relative entropy calculation and (ii) a streamlined assessment of the relative area and localization of the inclusions in the cell. The method has been successfully applied to several microalgal and cyanobacterial strains capable of accumulating PRIs and NRIs.

## Materials and methods

### Microalgal strains and cultivation conditions

The following strains served as models in this work: eukaryotic green microalgae *Chlorella vulgaris* IPPAS C-1 and *Desmodesmus* sp. IPPAS S-2014 earlier referred as *Desmodesmus* sp. 3Dp86E-1 (see [27,39]) and a cyanobacterium *Nostoc* sp. PCC 7118. The strains are referred to below as *C*. *vulgaris*, *Desmodesmus* sp.and *Nostoc* sp., respectively.

Pre-cultures of the microalgae were grown in 750 mL Erlenmeyer flasks (*C*. *vulgaris*, *Desmodesmus* sp.) or in glass columns (6 cm internal diameter, 1.5 L volume) (*Nostoc* sp.) in 400 mL of BG-11 medium [40] at 40 μmol PAR photons m^−2^ s^−1^ by a white light emitting diode source as measured by LiCor 850 quantum sensor (Licor, USA) with manual mixing (once a day for *C*. *vulgaris*, *Desmodesmus* sp.) or constant bubbling with a 2% CO_2_-air mixture (*Nostoc* sp.). The pre-cultures were kept at the exponential growth phase by daily dilution with the medium. To obtain the cells with different abundance of PRIs, the microalgae were subjected to P starvation with subsequent replenishment of P in the medium according to the earlier developed protocol [41].

### Electron microscopy

The microalgal cells sampled from the P-starved cultures and the cultures at the stationary growth phase after P re-feeding were prepared for transmission electron microscopy (TEM) according to the standard protocol: fixed in 2% v/v glutaraldehyde solution in 0.1 М sodium cacodylate buffer (pH 6.8–7.2, depending on the culture pH) at room temperature for 0.5 h and then post-fixed for 4 h in 1% (w/v) osmium tetroxide (ОsО_4_) in the same buffer. The samples, after dehydration through graded ethanol series including anhydrous ethanol, were embedded in araldite. Ultrathin sections were made with an LKB-8800 ultramicrotome (LKB, Sweden), mounted to the formvar coated TEM grids. The ultrathin sections were examined under JEM-2100 (JEOL, Japan) microscope equipped with a LaB_6_ gun at the accelerating voltage of 200 kV.

EELS analysis was performed on ultrathin sections using Gatan GIF Quantum ER spectrometer (Gatan, USA). EELS point spectra from the cellular inclusions were recorded in high-angle annular dark-field scanning transmission electron microscopy (HAADF-STEM) mode using Gatan 806 HAADF-STEM detector (Gatan, USA). The energy-loss range was 100–600 eV, which includes C, N, P, S, Ca, Cl, and O edges. Digital Micrograph software (Gatan, USA) was used for spectra processing. The background was approximated by a power-law function. N and P elemental mapping of whole cell ultrathin sections was carried out using EFTEM three-window method with a 15 eV energy selecting slit. Two pre-edge images at 371 eV, at 388 eV and one post-edge image at 412 eV were recorded for N mapping (CCD binning 4), and two pre-edge images at 104 eV, at 121 eV and post-edge image at 144 eV for P mapping (CCD binning 2). The energy windows were chosen based on the electron energy loss spectra to avoid overlap with other element edges. The objective aperture was 40 μM. The elemental distributions were calculated automatically using Digital Micrograph software (Gatan, USA) after alignment of the obtained images using a power law as the background model. Specimen thickness was controlled to ensure that it did not exceed the optimal value for EFTEM (below 0.5 λ, where λ is inelastic mean free path length for the chosen experimental conditions).

### The method of EFTEM map analysis

**Fig 1:**
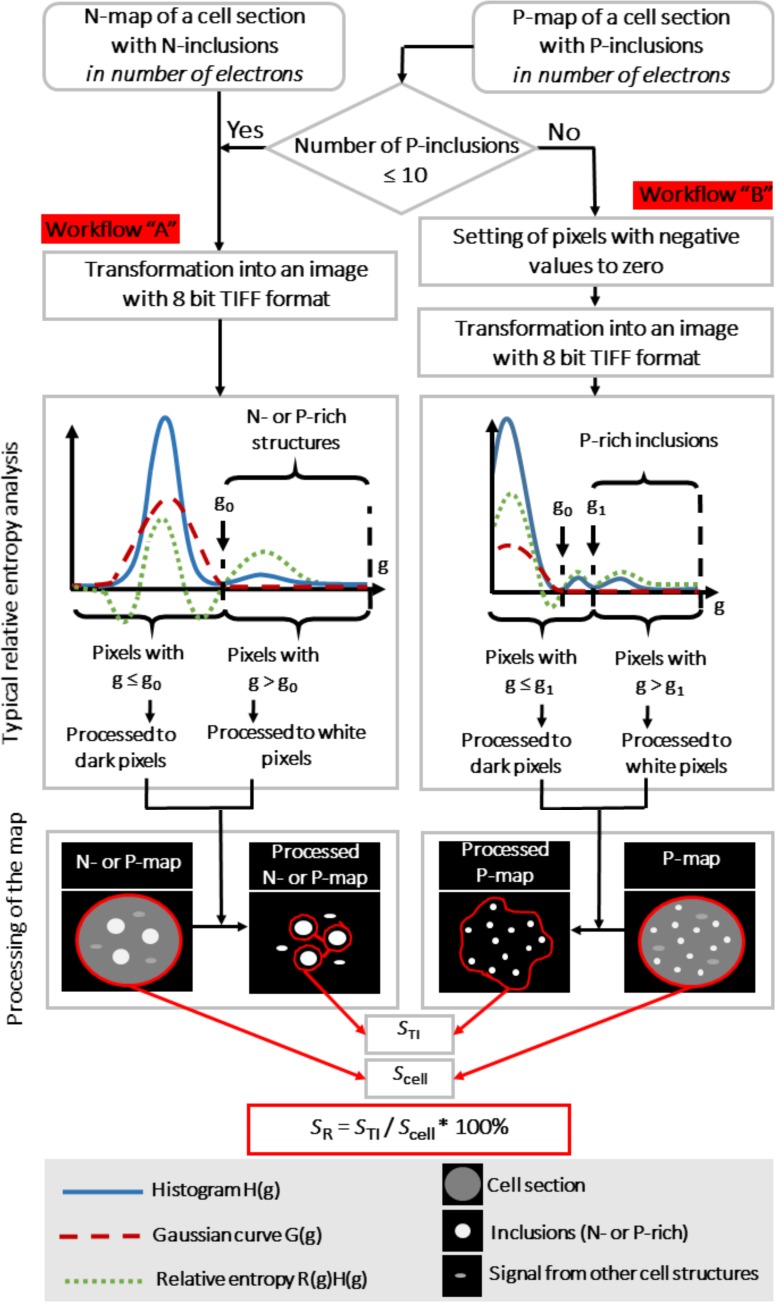
Scheme of the processing of EFTEM N- and P-maps. The method of EFTEM maps processing may be implemented *via* two alternative workflows. The workflow “A” (left column) is used for processing N-maps and low-phosphorus maps (less than 10 PRIs per cell section), the workflow “B” (right column) is used for processing P-maps with more than 10 PRIs per cell section. The workflows are based on comparison of the *a posteriori* probability that a map pixel contains payload information and the Gaussian *a priori* probability of that the pixel belongs to noise. The difference is expressed as relative entropy value for each pixel so pixels with positive relative entropy values grouped in the right half of the histogram do not belong to the noise. After the relative entropy analysis of each pixel and subsequent binary thresholding of the map, a simple assessment of the relative area and localization of the inclusions on the cell section is carried out. *S*_TI_−total pixel area of the cell inclusions, *S*_cell_−total pixel cell area, *S*_R_−relative area of P or N reserves. See text and S1 and S2 Appendices for details.

The method consists of two alternative workflows (Fig 1). The workflow selection is based on the type and amount of the inclusions of interest present on the EFTEM map under analysis. For mapping of nitrogen (N-maps) and -phosphorus maps for samples with a relatively low P content (less than 10 PRIs per cell section) the workflow “A” was used, for P-maps with more than 10 PRIs per cell section the workflow “B” was used (see Fig 1). The calculations were performed using Microsoft Excel spreadsheet software (Microsoft, USA).

Both map analysis workflows are based on comparison of the *a posteriori* probability that a map pixel contains payload information and the Gaussian *a priori* probability of that the pixel belongs to noise. The difference is expressed as relative entropy value for each pixel so pixels with positive relative entropy values grouped in the right half of the histogram do not belong to the noise. After the relative entropy analysis of each pixel and corresponding binary thresholding of the map, a simple assessment of the relative area and localization of the inclusions on the cell section is carried out.

The first step in both workflows was the transformation of the raw map (where each pixel represents the intensity of signal, i.e. number of electrons) into a 8-bit TIFF image. The transformation was carried out as follows: the pixels were mapped to levels of grey *via* a linear transformation as described by Trebbia and Mory [38] resulting in an image with 256 levels *g* (*g*_*min*_ = 0, *g*_*max*_ ≤ 255). The workflow “B” differed by additional step—setting all pixels of the map with negative values to 0 before the transformation to TIFF image (right column in Fig 1).

The second stage, common for the both workflows, involved the computing of the relative entropy value for each pixel of the resulting TIFF images (for more details, see [38]; for the rationale of the image processing algorithm see [37]). The histogram *H*(*g*) of the *a posteriori* probability, together with additional parameters, were calculated:

$$g_{mean}=\frac{1}{P}\sum gH(g),$$ (1)

$$\sigma^{2}=variance(H)=\frac{1}{P-1}\sum \left[{\left(g-g_{mean}\right)}^{2}H(g)\right],$$ (2)

where *P* = ∑*H*(*g*) is the total number of pixels in the map.

Then the Gaussian *a priori* probability *G*(*g*) was calculated for each grey level, *g*, of the map and relative entropy, *q*(*H*/*g*), of each pixel was estimated:

$$G(g)=\left(\frac{P}{\sqrt{2\pi \sigma^{2}}}\right)e^{\left[-{\left(g-g_{mean}\right)}^{2}/2\sigma^{2}\right]},$$ (3)

$$q\left(\frac{H}{G}\right)=\frac{1}{P}\sum R(g)H(g),$$ (4)

$$\mathrm{where}\;R(g)=\log\left[\frac{H(g)}{G(g)}\right].$$ (5)

The third step was constituted by plotting the histogram *H*(*g*), the Gaussian curve *G*(*g*) and the relative entropy, *R*(*g*)*H*(*g*), at the same graph (see Fig 1).

The grey levels (*g*) on the right half of the histograms corresponded to positive relative entropy values. It means that for such pixels the *a posteriori* probability, *H*(*g*), is greater than the Gaussian *a priori* probability *G*(*g*) under the same experimental conditions (i.e. same number of electrons incident on the same number of pixels) [37,38]. Considering P- and N-maps, this means that the pixels of the map with high *g* values (greater than *g*_0_) belong to the P- or N-rich cell structures, otherwise the pixels belongs to the noise. Essentially, this approach allows to distinguish the cell structures rich in P and/or N and pixels that belong to the noise.

The final step of the EFTEM map processing includes binary thresholding of maps using a contrast limit to classify each pixel of a map as white (i.e. containing a significant amount of the element of interest) or black (content of the element of interest is too low). In the workflow “A”, the pixels with *g* > *g*_0_ are white and the pixels with *g* ≤ *g*_0_ are black (see left column in Fig 1). In this case, the inclusion area was measured on the processed maps by manually selecting the structures to avoid including of the white pixels from other cell structures. This operation does not take much time since total number of the inclusions was < 10. The number of white pixels in the selected area was accepted as the pixel area of the inclusion, *S* (Fig 1). Finally, a total area of the cell inclusions, *S*_TI_, was calculated.

The workflow “B” dictates that the pixels with *g* > *g*_1_ are white and the pixels with *g* ≤ *g*_1_ are black, where *g*_1_ > *g*_0_ (see right column Fig 1). On the relative entropy curve of the P-maps, the value *g*_1_ was determined as the end of the first peak to the right of the *g*_0_. This peak corresponded to P-containing cell structures except the PRIs. The map pixels with *g* > *g*_1_ were considered as belonging to the PRIs. Area of the inclusions on the processed map was manually selected. The number of white pixels in the selected region was accepted as the total pixel area of the inclusions (*S*_TI_) (Fig 1).

Then total cell area, *S*_cell_, was calculated (in pixels) and the relative area of N or P reserves, *S*_R,_ in this cell was computed (see Fig 1):

$$S_{R}=\frac{S_{\mathrm{TI}}}{S_{cell}}*100\%$$ (6)

The *S*_cell_ and *S*_TI_ were measured on the EFTEM maps of random cell ultrathin sections (*n* = 18) and on the corresponded processed EFTEM map respectively, using ImageJ software (NIH, Bethesda MA, USA). The relative area of N- or P-reserve structures (*S*_R_; i.e. ratio of the inclusion area to the total area of the studied cell section) was measured on the random cell ultrathin sections (*n* = 18 for each sample).

For the measurement of *S*_TI_ of PRIs, the PRIs were located using survey TEM images of the cell sections. In the *Nostoc* sp. images, attributability to a poly-(R)-3-hydroxybutyrate (PHB)-rich structure was tested for each PRI. In the *C*. *vulgaris* and *Desmodesmus* sp. images, attributability to vacuolar, cytosol, chloroplast or nuclear localization were tested. The cases of uncertain localization of the PRIs were designated as “not determined”.

In the case used for this work, cell heterogeneity of the studied cultures was probed from 18 random cell sections for each sample. Measurement of *S*_R_ by the proposed method was repeated 3 times for each cell section (standard error was no more than 0.03% for each studied cell section).

## Results

### Application of the method to EFTEM maps

**Fig 2:**
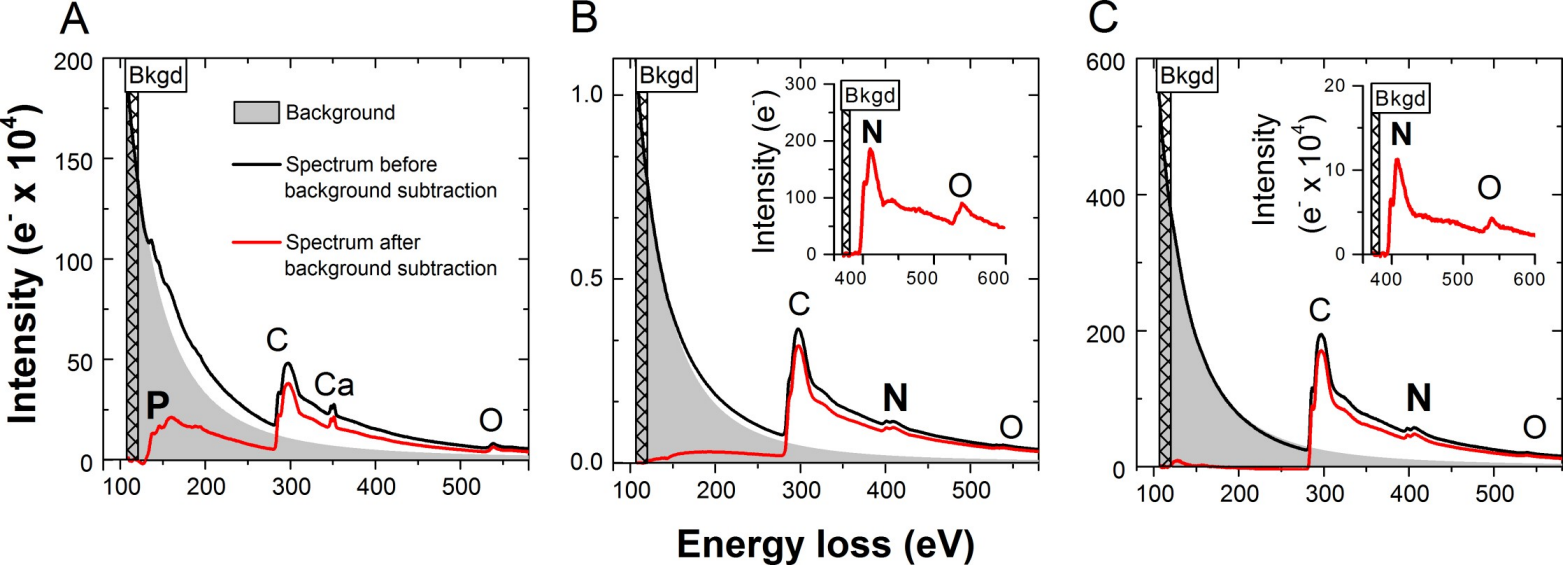
Typical EEL spectra of P-rich and N-rich inclusions. (A) Spectrum of P-rich inclusion in *Chlorella vulgaris* IPPAS C-1. (B) Spectrum of N-rich inclusion in *Chlorella vulgaris* IPPAS C-1. (C) Spectrum of N-rich inclusion in *Nostoc* sp. PCC 7118. The hatched rectangle in the EEL spectra indicates the range used for fitting of the power law function representing the background for subtraction. Inserts in (B) and (C): enlarged part of the same spectra background-corrected using the range just before the peak of N.

**Fig 3:**
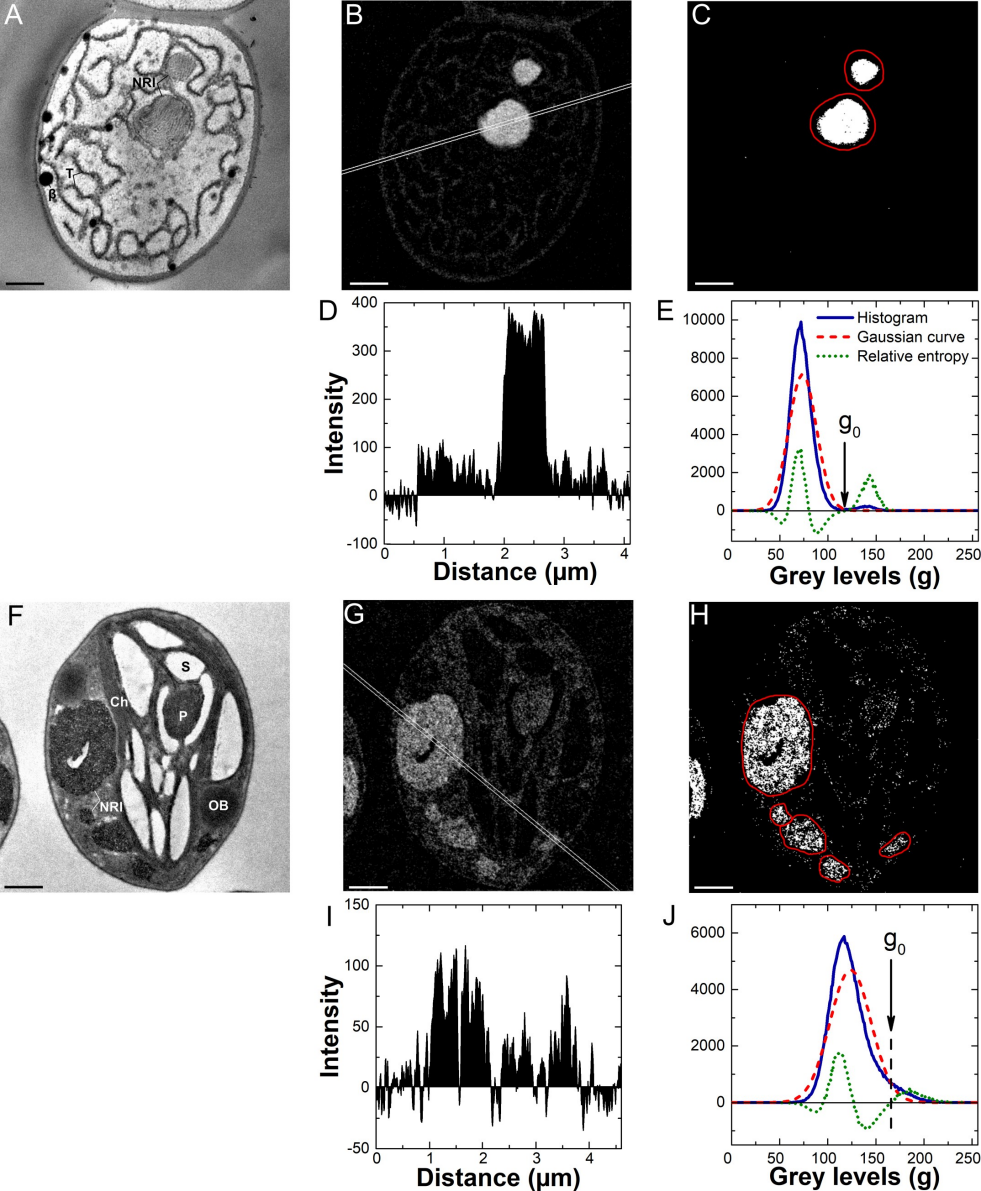
Application of the proposed method to the EFTEM N-maps. (A-E) The results of the method application to a cyanobacterium *Nostoc* sp. PCC 7118 from the P-starved culture. (F-J) The results of the method application to a eukaryotic microalga *Chlorella vulgaris* IPPAS C-1 from the P-starved culture. (A) and (F) Elastically filtered TEM images of cell sections. (B) and (G) EFTEM N-maps of the cell sections. (D) and (I) Averaged profiles of the EFTEM N-maps. (C) and (H) The EFTEM maps processed according to the workflow “A” (see text and Fig 1). (E) and (J) The relative entropy analysis of the EFTEM maps (B) and (G), respectively. The averaged profiles were recorded along the white lines (see the N-maps). The red outline on the processed maps (C) and (H) indicates the region taken for the inclusion area measurements. In the graphs (E) and (J) the threshold pixels *g*_0_ used for the EFTEM maps processing are designated (for details see text). *CG* cyanophycin granule, *T* thylakoid(s), *β* high electron density lipid β-granules, *Ch* chloroplast, *NRI* nitrogen-rich inclusion, *S* starch, *P* pyrenoid, *OB* oil body. Scale bars = 0.5 μm.

The proposed method was tested on the eukaryotic chlorophytes *C*. *vulgaris* and *Desmodesmus* sp. and a cyanobacterium *Nostoc* sp. cell section maps. Our preliminary studies yielded the EFTEM maps and EEL spectra similar to those showed in Figs 2 and 3 and S1 and S2 Figs suggesting that these microorganisms accumulated, under P starvation, NRIs. At the stationary growth phase following the re-feeding of the P-starved cells with inorganic phosphate the microalgal cells accumulated PRIs. The method was used for quantification of the NRIs in the P-starved cultures and PRIs in the P-sufficient cells sampled from the stationary cultures.

The typical EEL spectra of the PRI (Fig 2A) and NRI (Fig 2B) in *C*. *vulgaris* cells are shown together with the spectrum of NRI (Fig 2C) typical of the cyanobacterium *Nostoc* sp. The EEL spectra of the PRIs in *Desmodesmus* sp. (Fig A in S1 Fig) and *Nostoc* sp. (Fig B in S1 Fig) were similar to those in *C*. *vulgaris*. The EEL spectra of the NRIs in *Desmodesmus* sp. were similar to those of *C*. *vulgaris* except for the difference in the NRI signal level (Fig C in S1 Fig). The presence of P in the spectra taken from the PRIs was indicated by the characteristic L_2,3_-edge at an energy loss of 132 eV, which was most often accompanied by the L_2_-edge (350 eV) and L_3_-edge (346 eV) of calcium (Fig 2A, Fig B in S1 Fig). The presence of N in spectra from NRIs was indicated by the characteristic K-edge at an energy loss of 401 eV (Fig 2B and 2C, Fig C in S1 Fig). These spectra were used for the selection of the corresponding energy windows for the EFTEM mapping (see “Material and methods” section).

**Fig 4:**
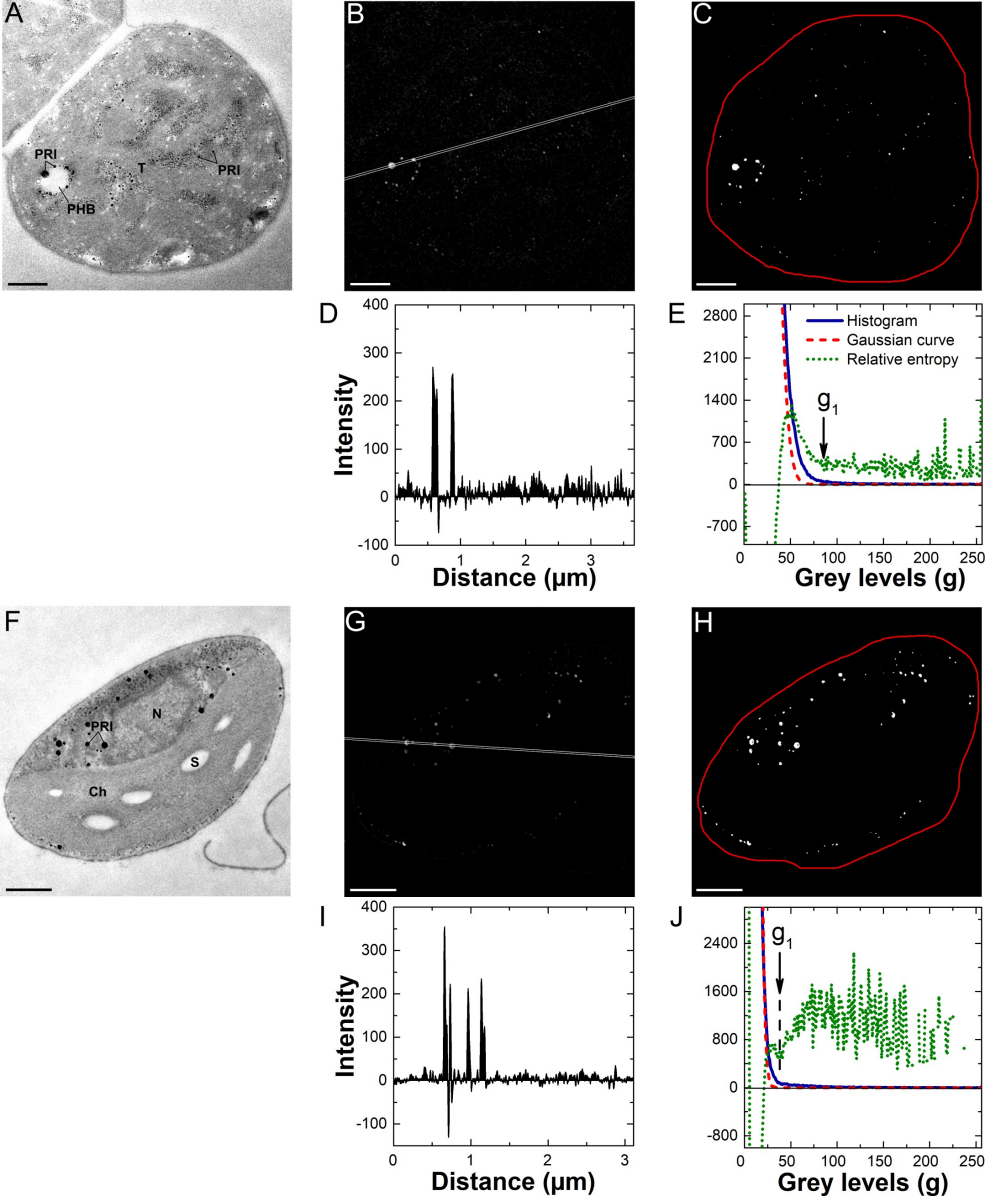
Application of the proposed method to the EFTEM P-maps. (A-E) The results of the method application to a cyanobacterium *Nostoc* sp. PCC 7118 from the P-sufficient stationary phase culture. (F-J) The results of the method application to a eukaryotic microalga *Chlorella vulgaris* IPPAS C-1 from the P-sufficient stationary phase culture. (A) and (F) Elastically filtered TEM images of cell sections. (B) and (G) EFTEM P-maps of the cell sections. (D) and (I) Averaged profiles of the EFTEM P-maps. (C) and (H) The EFTEM maps processed *via* the workflow “B” (see text and Fig 1). (E) and (J) The relative entropy analysis of the EFTEM maps (B) and (G), respectively. The averaged profiles were recorded along the white lines (see the P-maps). The red outline on the processed maps (C) and (H) indicates the region taken for the inclusion area measurements. In the graphs (E) and (J) the threshold pixels *g*_1_ used for the EFTEM maps processing are designated (for details see text). *PRI* P-rich inclusion, *PHB* poly-(R)-3-hydroxybutyrate granules, *T* thylakoid(s), *Ch* chloroplast, *N* nucleus, *S* starch. Scale bars = 0.5 μm.

The results of the analysis of *Desmodesmus* sp. cell section N or P EFTEM maps (S2 Fig) according to the workflow “A” or “B” (see “Material and methods” section and Fig 1) were similar to those obtained for *C*. *vulgaris* (Figs 3 and 4). The Excel spreadsheets illustrating the calculations behind the workflows “A” and “B” are available as S1 and S2 Appendices, respectively. The proposed method enabled the detection of tiny (<180 nm in diameter) PRIs and NRIs (Figs 3 and 4).

Abundance of the NRIs did not exceed 10 per cell section for the both eukaryotic microalgae and the cyanobacterium. For the analysis of the NRIs the workflow “A” was selected (Fig 3). When the workflow “B” was used for the quantification of these inclusions (S3 Fig), the relative entropy curves featured a single clearly pronounced peak belonging to the N-rich structures including NRIs. Therefore, it was not possible to separate signal derived from the NRIs and other N-rich structures. In the studied eukaryotic microalgae, the NRIs were localized in vacuoles (Fig 3F and 3G). The abundance of the PRIs in the studied microorganisms normally exceeded 10 per cell section, so the workflow “B” was used for their analysis (Fig 4).

When the workflow “A” was used for the quantification of the PRIs (S4 Fig), the inclusions on the processed maps were surrounded by the pixels belonging to irrelevant cell structures. Manual selection of multiple inclusions on such maps would be too time-consuming. However, zeroing of the negative values in the raw map before the processing (workflow “B”, see “Material and methods”) resulted in the formation of an additional peak on the entropy curve corresponding to the structures other than the structures of interest (right column in Fig 1, Fig 4E and 4J). In this case it was possible to automatically separate the signal from numerous inclusions and the signal from the irrelevant structures.

The inclusions of interest were readily apparent on the processed N- and P-maps (Fig 3C and 3H; Fig 4C and 4H) and their area, represented by white pixels, was easy to calculate by selecting the region containing these inclusions. The measured inclusion area was used for calculation of the relative area occupied by the inclusions (see “Material and methods”).

### Estimation of the N and P reserve abundance

**Fig 5:**
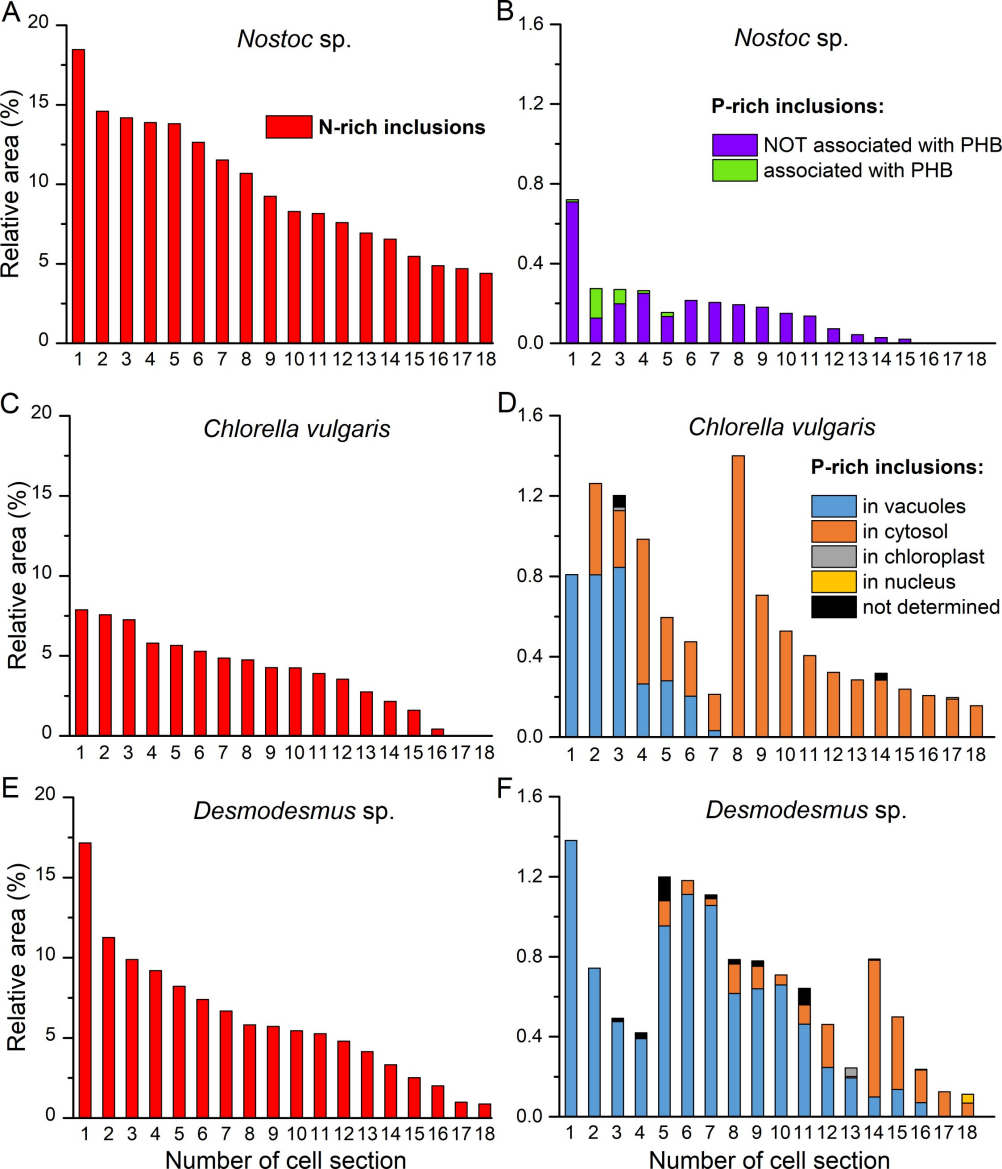
Estimation of the N and P reserve abundance in the studied cells. (A) and (B) Estimation of the reserves in the *Nostoc* sp. PCC 7118 cells. (C) and (D) estimation of the reserves in the *Chlorella vulgaris* IPPAS C-1 cells. (E) and (F) estimation of the reserves in the *Desmodesmus* sp. IPPAS S-2014 cells. (A), (C) and (E) estimation of the N reserves in the cells of P-starved cultures. (B), (D) and (F) estimation of the P reserves in P-sufficient cells sampled from the stationary phase cultures. The results expressed in relative area of the P-rich or N-rich inclusions are shown for each cell section (*n* = 18). Relative area values on the histograms are sorted in descending order (A), (C), (E) or in descending order within the following groups: in (B), both P reserves associated with PHB and P reserves not associated with PHB; only P reserves not associated with PHB; in (D) and (F), main P reserves in the vacuoles, no P reserves in the cytosol; P reserves both in the vacuoles and in the cytosol and the main P reserves are located in the vacuoles; P reserves both in the vacuoles and in the cytosol and the main P reserves are located in the cytosol; the main P reserves in the cytosol, no P reserves in the vacuoles. For the P-rich inclusions (B), (D), (F) subcellular localization is also shown.

Relative inclusion area for NRIs or PRIs was calculated for each cell section of *Nostoc* sp., *C*. *vulgaris* and *Desmodesmus* sp. (Fig 5). Relative area of NRIs for *Nostoc* sp., *C*. *vulgaris* and *Desmodesmus* sp. was in the range 4.4%–18.5% (Fig 5A), 0–7.9% (Fig 5C), and 0.9–17.2% (Fig 5E), respectively.

Abundance of the NRIs in *C*. *vulgaris* cells was the lowest among the studied microorganisms: their relative area did not exceed 8%, two of 18 studied cell sections did not contain NRIs at all (Fig 5C). The cells of *Nostoc* sp. harbored more NRIs than the *Desmodesmus* sp. cells: 11 of 18 cell sections of *Nostoc* sp. possessed the NRIs occupying more than 8% of the section area (Fig 5A) while only 5 of 18 cells of *Desmodesmus* sp. featured the presence of the NRIs occupying more than 8% of the section area (Fig 5E).

Generally, the relative area of the PRIs was lower than that of the NRIs in all studied microorganisms. The abundance of the PRIs per cell section did not exceed 0.8% for *Nostoc* sp. (Fig 5B) or 1.4% for *C*. *vulgaris* (Fig 5D) and *Desmodesmus* sp. (Fig 5F). The lower relative area of the PRIs meant the lower number of pixels classified as belonging to this inclusion type in comparison with NRIs; it can be a plausible reason for the absence of a clearly defined peak corresponding to the PRIs on the relative entropy curves (see Fig 5L and 5P).

The cells of *Nostoc* sp. contained more PRIs in comparison with other studied microalgae: only three of 18 studied cell sections did not contain PRIs (Fig 5B).

The proposed method enabled us to classify the PRIs of *Nostoc* sp. cells as associated or not associated with PHB (Fig 5B): five of 18 studied cell sections possessed the PRIs associated with PHB, only in one of the cell sections the main P reserves were in PHB-associated PRIs, in other cell sections the main P reserves were localized in the PRIs not associated with PHB.

The main P reserves in the *C*. *vulgaris* and *Desmodesmus* sp. cells were in the PRIs localized in the vacuoles or cytosol (Fig 5D and 5F). The PRIs situated in the chloroplast, nucleus and the inclusions with undetermined localization occupied a much smaller part of the cell area (< 0.06% and 0.13% for *C*. *vulgaris* and *Desmodesmus* sp., respectively; Fig 5D and 5F).

**Fig 6:**
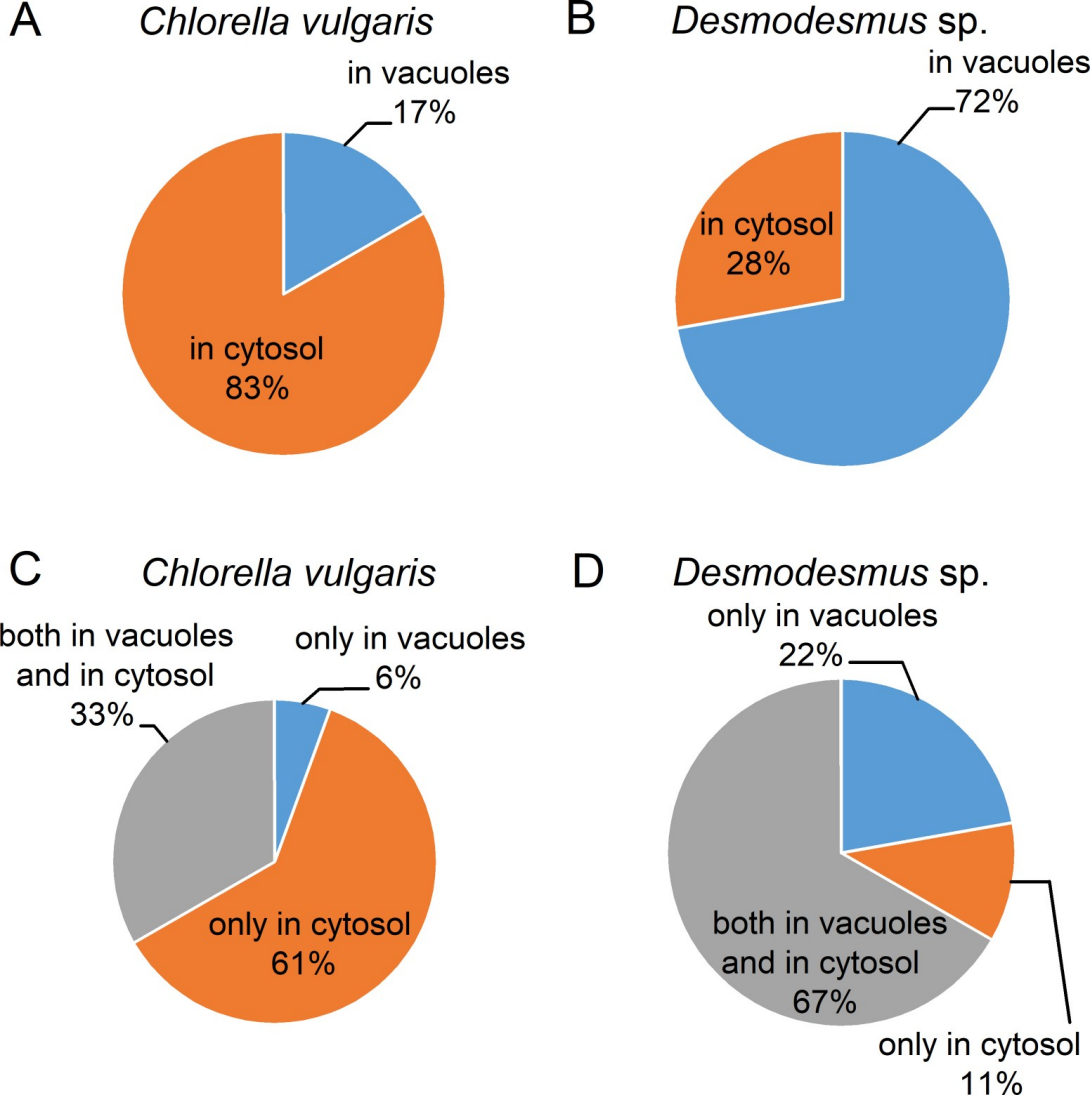
Localization of main P reserves and total P reserves in the studied microalgae. The results are represented for cells sampled from the P-sufficient stationary phase cultures. (A) and (B) Main P reserves in *Chlorella vulgaris* IPPAS C-1 and *Desmodesmus* sp. IPPAS S-2014, respectively. (C) and (D) Total P reserves in *Chlorella vulgaris* IPPAS C-1 and *Desmodesmus* sp. IPPAS S-2014, respectively. Percentages of cell sections containing the P reserves of the total number of studied cell sections are shown. In (C) and (D) the inclusions localized in chloroplast, nucleus and the inclusions with undetermined localization were not taken into account.

We also compared the localization of the main and total P reserves in *C*. *vulgaris* and *Desmodesmus* sp. cells (Fig 6). Most of the *C*. *vulgaris* cell sections possessed the main P reserves in cytosol (Fig 6A), while most of the *Desmodesmus* sp. cell sections–in vacuoles (Fig 6B).

The subcellular localization of the total P reserves in the cells of studied eukaryotic microalgae was also different (see Fig 6C and 6D): most of the *C*. *vulgaris* cell sections possessed the inclusions only in cytosol (Fig 6C), whereas most of the *Desmodesmus* sp. cell sections harbored the inclusions both in the vacuoles and in the cytosol (Fig 6D).

## Discussion

A conventional approach to the evaluation of ultrastructural changes in eukaryotic microalgae and cyanobacteria including changes in PRIs and NRIs abundance is constituted by direct morphometry of the cell section TEM images [14,42–48]. This type of quantification is carried out manually hence it is labor-intensive and error-prone. Even more serious is the problem of reliable identification of the structure type which is hard to achieve using survey TEM images without objective information on the elemental composition of the structures. In contrast, the method proposed in this study offers the analysis of EFTEM maps according to well-defined criteria taking into account the objective data on the elemental composition of the structures of interest. Remarkably, implementation of this method does not require as much time as the conventional TEM-based morphometry because it is based on automatic binary thresholding of the EFTEM maps yielding a bitmap. This kind of processing makes possible a simplified area-based quantification of the analyzed structures (e.g., PRIs or NRIs) by summation of the white pixels attributed to these structures in the selected region of the map.

In our study, for each pixel of the N- or P-map the contrast limit of the map was set similarly to the routines described in Koop et al. [26] who used binary thresholding of EFTEM N-maps to identify and localize the cyanophycin granules in recombinant strains of *Ralstonia eutropha*. In that work [26], pixels with a signal-to-noise-ratio (SNR) ≥ 3 were considered N-containing (white) if it was known in advance that the sample contains N, otherwise the pixels with a SNR ≥ 5 were considered N-containing. The SNR for each pixel was calculated by standard procedures [49] using the post-edge image and pre-edge images. These approaches are calculation-intensive, their practical implementation is difficult for biologists lacking a professional background in mathematics and statistics [49]. In the present study we suggest processing of EFTEM maps *via* simple calculations based on relative entropy which do not require advanced knowledge of statistics and can be implemented in standard spreadsheets like Excel (see S1 and S2 Appendices). Specifically, the relative entropy is inferred directly from the EFTEM maps after conventional background subtraction eliminating the raw post- and pre-edge image manipulations necessary for the SNR-based method [26].

The P reserves (mainly PolyP in biological samples) can be assayed using chemical analysis, chromogenic tests, enzymatic methods, 4′,6-diamidino-2-phenylindole (DAPI) staining-based fluorometric methods, ^31^P nuclear magnetic resonance spectroscopy and Raman microscopy [50,51]. All these methods except Raman microscopy allow to analyze only bulk cell suspension samples yielding an average PolyP abundance in the cell population or at a single cell level if a flow cytometer is used. A key advantage of the proposed method is its subcellular resolution. A method based on Raman microscopy [41] also gives information about intracellular distribution of PolyP and, as the proposed method, allows to assess heterogeneity of individual cells [41]. However, the using of confocal microscope limits its spatial [52] resolution to roughly 180 nm which is insufficient to resolve the intracellular (cytosolic, vacuolar, chloroplast, nuclear, mitochondrial etc.) inclusions [41]. The method proposed in this study does not suffer from these limitations.

The selection of methods for quantification of cyanophycin in cyanobacteria is even scarcer: the standard method based on a chemical assay for arginine [53] and a more rapid and sensitive method based on ^1^H nuclear magnetic resonance (NMR) spectroscopy [54]. Both methods [53,54] analyze the isolated cyanophycin granules and provide an averaged value for the bulk sample whereas our method allows quantification of cyanophycin with subcellular resolution. Notably, cyanophycin granules from dead and destroyed cells retain their integrity [47] and interfere with the arginine-based assay [53] and ^1^H NMR spectroscopy [54]. The method proposed in the study allows to estimate cyanophycin granules only in the cells that were alive at the moment of fixation for TEM and retained their integrity judging from their morphological features on the cell sections.

The literature available at the time of this research lacked reports on quantitative analysis of NRIs in eukaryotic microalgal cells (represented by guanine and/or uric acid). It is likely that such a method for quantification of guanine using Raman microscopy will be developed [15] as it was done earlier for PolyP quantification [41] but the spatial resolution of Raman microscopy is limited (see above). Therefore, the method proposed here is, to the best of our knowledge, the first method for simple quantification and location of P-rich and N-rich structures including small ones (< 180 nm) at subcellular level.

Limitations of the method proposed in the study generally stem from the requirement of ultrathin cell sections for the EFTEM analysis. First, one should remember that a cell section is not equal to whole cell volume and a single EFTEM map visualizes only the content of a single section of the cell. Second, this method provides information only on the area occupied by inclusions which contain enough P or N atoms sufficient for detection by the EFTEM. The method does not take into account the difference in the inclusion density or magnitude of the signal from them. In view of what is said above, this method is most suitable for comparative assessments of P and N reserve abundance. For absolute quantification, other available quantitative methods should be used [41,50,53–57]. Confirmation of chemical nature of the inclusions by independent methods (NMR spectroscopy, Raman microscopy, etc.) is highly advisable. Third, extremely small inclusions (mainly PRIs) might not be detectable with EFTEM. Fourthly, one should be cautious of the inclusion matter crumbling out of the sections during their preparation (especially frequent in vacuolar inclusions). And finally, sometimes it is difficult to determine reliable localization of tiny P-inclusions (e.g. in cytosol or in a small vacuole). Most often this difficulty results from poor contrast of membranes because of exclusion of uranyl acetate staining from the sample preparation for the EFTEM P-mapping. This is required to avoid the interference from the overlap of the uranium peak and the range of the EEL spectra before P peak which is used for estimation the background in the EFTEM P-mapping [27]. We believe that most of the abovementioned limitations could be overcome by working with a sufficiently large set of cell EFTEM maps.

In our work, we assessed the relative area of P and N reserves from the cell section EFTEM maps. A cell section is not equal to whole cell volume and a single EFTEM map visualizes only the content of a single section of the cell. Therefore, these assessments are comparable only with cell section-based data e.g. morphometry analysis of the P and N reserve structures in conventional TEM images.

With regard to TEM-morphometry analysis of PRIs in cyanobacteria, the relative area of PolyP inclusions in P-sufficient cyanobacteria *Anabaena variabilis* UTEX 1444 and *Nostoc muscorum* UTEX 1037 closely related to *Nostoc* sp. PCC 7118 is 1.6 and 0.4%, respectively [48]. P-sufficient cyanobacterium *Nostoc* sp. PCC 7118 studied in our work is capable of more intensive accumulation of PRIs than *N*. *muscorum* UTEX 1037 in the work of Lawry and Simon [48] (up to 0.8% versus 0.4%), but of less intensive accumulation than *A*. *variabilis* UTEX 1444 in the work of Lawry and Simon [48] (up to 0.8% versus 1.6%).

The relative area of NRIs in P-sufficient cyanobacterium *Nostoc muscorum* CALU 304 calculated from the data published in the work of Korzhenevskaya et al. [45] is 5–6%. The results [45] for relative area of NRIs correspond to our results for *Nostoc* sp. PCC 7118 (Fig 5A) in order of magnitude. According to [48] the relative area of NRIs in P-deficient cyanobacteria *A*. *variabilis* UTEX 1444 and *N*. *muscorum* UTEX 1037 is 10.2 and 5.4%, respectively. P-deficient cyanobacterium *Nostoc* sp. PCC 7118 studied in our work is capable of more intensive accumulation of NRIs: by almost 2 times more than *A*. *variabilis* UTEX 1444 in the work of Lawry and Simon [48] (up to 18.5% versus 10.2%), and by more than 3 times in comparison with *N*. *muscorum* UTEX 1037 in the work of Lawry and Simon [48] (up to 18.5% versus 5.4%).

We have not found published data of such type for PRIs in eukaryotic microalgae, although the relative area of NRIs in microalga *Symbiodium* sp. (designated as uric acid crystals) living in symbiosis with coral host under phosphorus limitation did not exceed 4% basing on 100 examined cell sections [14]. P-deficient microalgae *C*. *vulgaris* and *Desmodesmus* sp. studied in our work are capable of more intensive accumulation of NRIs than *Symbiodium* sp. in the work of Rosset et al. [14]: the relative area of the NRIs in the *C*. *vulgaris* and *Desmodesmus* sp. can reach 7.9% and 17.2%, respectively.

The proposed simplified method of the EFTEM map processing should be handy for cytological and microbiological studies or, generally, in all fields where (comparative) assessments of subcellular distribution of cyanophycin, PolyP and other nutrient-rich inclusions are needed. One of the illustrious examples is the study of the effect of cultivation on subcellular distribution of guanine and PolyP in microalgae. The information about the distribution of N and P among the cell (sub)compartments is a unique outcome of this method. It will be crucial for obtaining a deeper mechanistic insight into luxury uptake of phosphorus and for dissecting the effects of silencing and/or overexpressing genes involved in the regulation and implementation of nutrient storage in the cell. We assume that this technique is potentially applicable to other types of microorganisms or cells capable of accumulating PRIs and/or NRIs. The main prerequisites for the application of this method include: (i) sufficient amount of P and/or N atoms within the inclusions for detection by the EFTEM, and (ii) possibility to obtain an EFTEM map encompassing the entire cell section to estimate their area.

The subdivision of the method into two workflows made it possible to analyze both cell sections with small (the workflow “A”) and large (the workflow “B”) number of inclusions. The workflow “A” involving all pixels with positive values of relative entropy is problematic when applied to the cell sections with a large number of the inclusions (S4 Fig). Ten inclusions per section was accepted as the threshold value for the choice between the workflows. Namely, up to 10 inclusions/cell section can be quickly identified on maps with the workflow “A”. In the workflow “B”, negative values of pixels in the raw maps are set to zero. As a result, an additional peak corresponding to the structures other than the structures of interest appeared in the entropy curve of the maps with a large number (>10) of inclusions. This feature likely arising from pixel zeroing was used for separation of the signal of the inclusions. However, the workflow “A” applied for the maps did not yield this peak (S4 Fig). This peak also lacked when the workflow “B” was applied to maps with a small number of inclusions (≤10) (S3 Fig). We believe that it was due to a lower relative area of the inclusions typical of the maps with numerous inclusions (>10) but unusual for the map with a small number of inclusions (≤10). Hence these maps yielded the relative entropy curves lacking a clearly defined peak corresponding to the inclusions, so the peak corresponding to the irrelevant structures became visible.

Importantly, almost all stages of both workflows constituting the method (excepting manual selection of the inclusion area in the workflow “A” and the cell area calculation in both workflows) have potential for automatization. In view of this, the proposed method is the first approach supporting semi-automatic EFTEM map processing and evaluation. Upon implementation of this capacity, our approach will increase significantly the throughput to EFTEM mapping. In our study we demonstrated the feasibility of the cell P and N reserve heterogeneity estimation with this method in the studied microorganisms. In our work the cell heterogeneity was probed from 18 random cell sections for each sample. Therefore, standard statistical treatment of the data was inappropriate for revealing the biological effect, although it does not undermine the validity of the EFTEM map analysis. Repeated measurements (*n* = 3 for each cell section) by the proposed method for the same cell section showed no significant differences (standard error was no more than 0.03% for each studied cell section). So, the technique itself yields reproducible results. At the same time, it is important to understand that to enable the calculation of standard statistics like means and standard deviations for comparison of control and experimental groups one just needs to feed a sufficiently large set of cell EFTEM maps to this method. For example, the standard statistics were calculated in *Symbiodinium* sp. for the relative NRIs area dataset obtained by processing 100 cell sections for each sample (see [14]). By the way, this highlights the other potential strength of our method (possibility of automated analysis of the EFTEM maps to acquire the large datasets required for conventional statistics analysis more easily).

We also revealed peculiarities of the P and N reserves accumulation in two chlorophyte species grown under similar conditions, and the cyanobacterium *Nostoc* sp. With regard to NRIs, we found out that the signal derived from the NRIs of *C*. *vulgaris* was significantly lower than the signals derived from the inclusions of *Desmodesmus* sp. and *Nostoc* sp. (Fig 2B and 2C, Fig C in S1 Fig), most likely due to differences in their density and fine structure of the NRIs.

We also determined the localization of the P reserves both in cyanobacterial and in microalgal cells. We were able to spatially resolve the contrasting types of PRIs in the *Nostoc* sp. cells (PHB-associated or not associated with PHB). Colocalization of PolyP and PHB is important in the studies of PolyP formation and deposition in microalgae since we assumed, basing on our previous results [27], that PolyP chains constituting the PRI in vacuoles of eukaryotic microalgae might be embedded into the PHB matrix. Hence the colocalization of PolyP and PHB in cyanobacterial cells is potentially indicative of different biosynthetic pathways PolyP as well as in microalgal cells. We showed that in the studied microalgal strains *C*. *vulgaris* and *Desmodesmus* sp., the main P reserves were in the form of the vacuolar and/or cytosolic PRIs, whereas the PRIs situated in the chloroplast, nucleus and the inclusions with unclear localization apparently made a minor contribution to the total cell P storage (Fig 5D and 5F). We also revealed the difference in the localization of different P pools in the two strains of green microalgae grown under the similar conditions.

## Conclusions

We propose a simplified method of EFTEM cell section map analysis for assessing the abundance and localization of P and N reserves in microalgal cells. Apart of simplicity and reliability, this method is characterized by a high spatial resolution (better than 180 nm). We demonstrated its applicability to the different cells including eukaryotic chlorophytes and cyanobacteria. Limitations of the method were discussed stemming mainly from the requirement of ultrathin cell sections for the EFTEM. Despite the limitations, the proposed method seems to be a powerful tool which can be used to quantify and to localize P and N reserves at the subcellular level. An added value of the proposed approach is its potential for semi-automation of the data processing and evaluation adding the high throughput capability to EFTEM which have not been considered before. The proposed technique can be potentially useful to estimate P or N reserves in other types of microorganisms or cells capable of accumulating PRIs or NRIs.

## Supporting information

S1 Appendix Demo Excel spreadsheet for the workflow “A”. Data generated for the N-map of *Nostoc* sp. PCC 7118 (Fig 3A–3E, cell section No.17 in Fig 5A) are used in the demo spreadsheet. The input data generated by ImageJ and imported into the spreadsheet (see “Material and methods”) is marked in green.
(ZIP)

S2 Appendix Demo Excel spreadsheet for the workflow “B”. Data generated for the P-map of *Nostoc* sp. PCC 7118 (Fig 4A–4E, cell section No.2 in Fig 5B) are used in this spreadsheet. The input data generated by ImageJ and imported into the spreadsheet (see “Material and methods”) is marked in green.
(ZIP)

S1 Fig Typical EEL spectra of P-rich and N-rich inclusions. (A) Spectrum of P-rich inclusion in *Desmodesmus* sp. IPPAS S-2014. (B) Spectrum of P-rich inclusion in *Nostoc* sp. PCC 7118. (C) Spectrum of N-rich inclusion in *Desmodesmus* sp. IPPAS S-2014. The hatched rectangle in the EEL spectra indicates the range used for fitting of the power law function representing the background for subtraction. Insert in (C): enlarged part of the same spectrum background-corrected using the range just before the peak of N.
(TIF)

S2 Fig Application of the proposed method to the EFTEM maps of *Desmodesmus* sp. IPPAS S-2014. (A-E) Application to N-map of cell from P-starved culture. (F-J) Application to P-map of cell from P-sufficient stationary phase culture. (A) and (F) Elastically filtered TEM images of cell sections. (B) and (G) EFTEM maps of the cell sections. (D) and (I) Averaged profiles of the EFTEM maps. (C) and (H) The EFTEM maps processed according to the workflow “A” and “B”, respectively (see text and Fig 1). (E) and (J) The relative entropy analysis of the EFTEM maps (B) and (G), respectively. The averaged profiles were recorded along the white lines (see the maps). The red outline on the processed maps (C) and (H) indicates the region taken for the inclusion area measurements. In the graphs (E) and (J) the threshold pixels *g*_0_ and *g*_1_, respectively, used for the EFTEM maps processing are designated (for details see text). *Ch* chloroplast, *N* nucleus, *NRI* nitrogen-rich inclusion, *OB* oil body, *P* pyrenoid, *PRI* P-rich inclusion, *S* starch, *V* vacuole. Scale bars = 0.5 μm.
(TIF)

S3 Fig Application of the workflow “B” to the EFTEM N-maps. (A) The results of the workflow application to a cyanobacterium *Nostoc* sp. PCC 7118 from the P-starved culture. (B) The results of the workflow application to a eukaryotic microalga *Chlorella vulgaris* IPPAS C-1 from the P-starved culture. (A) and (B) The relative entropy analysis *via* the workflow “B” of the EFTEM N-maps from Fig 3B and 3G, respectively. In the relative entropy curves there is one clearly pronounced peak belonging to the N-rich structures including N-rich inclusions.
(TIF)

S4 Fig Application of the workflow “A” to the EFTEM P-maps. (A) and (B) The results of the workflow application to a cyanobacterium *Nostoc* sp. PCC 7118 from the P-sufficient stationary phase culture. (C) and (D) The results of the workflow application to a eukaryotic microalga *Chlorella vulgaris* IPPAS C-1 from the P-sufficient stationary phase culture. (A) and (C) The EFTEM maps processed according to the workflow “A” (see text and Fig 1). (B) and (D) The relative entropy analysis *via* the workflow “A” of the EFTEM P-maps from Fig 4B and 4G, respectively. In the graphs (B) and (D) the threshold pixels *g*_0_ used for the EFTEM maps processing are designated (for details see text). The P-rich inclusions on the processed maps (A) and (C) are surrounded by the pixels belonging to other structures. Scale bars = 0.5 μm.
(TIF)

## Abbreviations

EELS: electron energy loss spectroscopy
EFTEM: energy-filtered transmission electron microscopy
HAADF-STEM: high-angle annular dark-field scanning transmission electron microscopy
N: nitrogen
NMR: nuclear magnetic resonance
NRI(s): nitrogen-rich inclusion(s)
P: phosphorus
PHB: poly-(R)-3-hydroxybutyrate
PolyP: polyphosphate(s)
PRI(s): P-rich inclusion(s)
SNR: signal-to-noise ratio
TEM: transmission electron microscopy
Nomenclature: *G*(g): Gaussian *a priori* probability
*H*(g): *a posteriori* probability
*R*(g)*H*(g): relative entropy
S: pixel area of inclusion
*S*_TI_: total pixel area of the cell inclusions
*S*_cell_: total pixel cell area
*S*_R_: relative area of P or N reserves

## References

[1] Abdel-Raouf N, Al-Homaidan AA, Ibraheem IB. Microalgae and wastewater treatment. Saudi journal of biological sciences. 2012;19: 257–275. 10.1016/j.sjbs.2012.04.005 24936135 10.1016/j.sjbs.2012.04.005 PMC4052567

[2] Mulbry W, Westhead EK, Pizarro C, Sikora L. Recycling of manure nutrients: use of algal biomass from dairy manure treatment as a slow release fertilizer. Bioresource technology. 2005;96: 451–458. 10.1016/j.biortech.2004.05.026 15491826 10.1016/j.biortech.2004.05.026

[3] Solovchenko A, Verschoor AM, Jablonowski ND, Nedbal L. Phosphorus from wastewater to crops: An alternative path involving microalgae. Biotechnology advances. 2016;34: 550–564. 10.1016/j.biotechadv.2016.01.002 26795876 10.1016/j.biotechadv.2016.01.002

[4] Bar-Yosef Y, Sukenik A, Hadas O, Viner-Mozzini Y, Kaplan A. Enslavement in the water body by toxic *Aphanizomenon ovalisporum*, inducing alkaline phosphatase in phytoplanktons. Current biology. 2010;20: 1557–1561. 10.1016/j.cub.2010.07.032 20705465 10.1016/j.cub.2010.07.032

[5] Martin P, Dyhrman ST, Lomas MW, Poulton NJ, Van Mooy BA. Accumulation and enhanced cycling of polyphosphate by Sargasso Sea plankton in response to low phosphorus. Proceedings of the National Academy of Sciences. 2014;111: 8089–8094. 10.1073/pnas.1321719111 PMC4050623 24753593

[6] Gobler CJ, Burkholder JM, Davis TW, Harke MJ, Johengen T, Stow CA, et al The dual role of nitrogen supply in controlling the growth and toxicity of cyanobacterial blooms. Harmful Algae. 2016;54: 87–97. 10.1016/j.hal.2016.01.010 28073483 10.1016/j.hal.2016.01.010

[7] Cembella AD, Antia NJ, Harrison PJ. The utilization of inorganic and organic phosphorous compounds as nutrients by eukaryotic microalgae: A multidisciplinary perspective: Part I. CRC Critical Reviews in Microbiology. 1982;10: 317–391. 10.3109/10408418209113567 6321101

[8] Kuhl A. Phosphorus In: Stewart WDP, editor. Algal Physiology and Biochemistry. Oxford: Blackwell Scientific; 1974 pp.636–654.

[9] Allen MM. Cyanobacterial cell inclusions. Annual Reviews in Microbiology. 1984;38: 1–25. 10.1146/annurev.mi.38.100184.000245 6437321

[10] Nishikawa K, Machida H, Yamakoshi Y, Ohtomo R, Saito K, Saito M, et al Polyphosphate metabolism in an acidophilic alga *Chlamydomonas acidophila* KT-1 (Chlorophyta) under phosphate stress. Plant Science. 2006;170: 307–313.

[11] Clode PL, Saunders M, Maker G, Ludwig M, Atkins CA. Uric acid deposits in symbiotic marine algae. Plant, cell & environment. 2009;32: 170–177. 10.1111/j.1365-3040.2008.01909.x 19021889

[12] Kopp C, Pernice M, Domart-Coulon I, Djediat C, Spangenberg JE, Alexander DT, et al Highly dynamic cellular-level response of symbiotic coral to a sudden increase in environmental nitrogen. MBio. 2013;4: e00052–13. Available from: http://mbio.asm.org/content/4/3/e00052-13.abstract 10.1128/mBio.00052-13 23674611 10.1128/mBio.00052-13 PMC3656441

[13] Raina JB, Clode PL, Cheong S, Bougoure J, Kilburn MR, Reeder A, et al Subcellular tracking reveals the location of dimethylsulfoniopropionate in microalgae and visualises its uptake by marine bacteria. eLife. 2017;6: e23008 Available from: https://elifesciences.org/articles/23008 10.7554/eLife.23008 28371617 10.7554/eLife.23008 PMC5380433

[14] Rosset S, Wiedenmann J, Reed AJ, D'Angelo C. Phosphate deficiency promotes coral bleaching and is reflected by the ultrastructure of symbiotic dinoflagellates. Marine pollution bulletin. 2017;118: 180–187. 10.1016/j.marpolbul.2017.02.044 28242282 10.1016/j.marpolbul.2017.02.044 PMC5441187

[15] Moudříková Š, Nedbal L, Solovchenko A, Mojzeš P. Raman microscopy shows that nitrogen-rich cellular inclusions in microalgae are microcrystalline guanine. Algal Research. 2017;23: 216–222.

[16] DeSa R, Hastings JW. The characterization of scintillons: Bioluminescent particles from the marine dinoflagellate, *Gonyaulax polyedra*. The Journal of general physiology. 1968;51: 105–122. 5642469 10.1085/jgp.51.1.105 PMC2201157

[17] Schmitter RE. The fine structure of *Gonyaulax polyedra*, a bioluminescent marine dinoflagellate. Journal of cell science. 1971;9: 147–173. 5565060 10.1242/jcs.9.1.147

[18] Fogel M, Schmitter RE, Hastings JW. On the physical identity of scintillons: bioluminescent particles in *Gonyaulax polyedra*. Journal of cell science. 1972;11: 305–317. 4341991 10.1242/jcs.11.1.305

[19] Egerton RF. Electron Energy-Loss Spectroscopy in the Electron Microscope. 2st ed. New York: Plenum Press; 1996.

[20] Reimer L. Electron spectroscopic imaging In: Reimer L, editor. Energy-Filtering Transmission Electron Microscopy. Springer Series in Optical Sciences, vol 71 Springer, Berlin, Heidelberg; 1995 pp. 347–400.

[21] Leapman RD. Novel techniques in electron microscopy. Current opinion in neurobiology. 2004;14: 591–598. 10.1016/j.conb.2004.08.004 15464893 10.1016/j.conb.2004.08.004

[22] Lütz-Meindl U. Use of energy filtering transmission electron microscopy for image generation and element analysis in plant organisms. Micron. 2007;38: 181–196. 10.1016/j.micron.2006.03.017 16766193 10.1016/j.micron.2006.03.017

[23] Aronova MA, Leapman RD. Development of electron energy-loss spectroscopy in the biological sciences. MRS bulletin. 2012;37: 53–62. 10.1557/mrs.2011.329 23049161 10.1557/mrs.2011.329 PMC3465455

[24] Albertano P, Canini A, Caiola MG. Sub-cellular distribution of nitrogen compounds in *Azolla* and *Anabaena* by ESI and EELS analysis. Protoplasma. 1993;173: 158–169.

[25] Jäger KM, Johansson C, Kunz U, Lehmann H. Sub‐cellular element analysis of a Cyanobacterium (*Nostoc* sp.) in symbiosis with *Gunnera manicata* by ESI and EELS. Plant Biology. 1997;110: 151–157.

[26] Koop A, Voss I, Thesing A, Kohl H, Reichelt R, Steinbüchel A. Identification and localization of cyanophycin in bacteria cells via imaging of the nitrogen distribution using energy-filtering transmission electron microscopy. Biomacromolecules. 2007;8: 2675–2683. 10.1021/bm0611230 17713945 10.1021/bm0611230

[27] Shebanova A, Ismagulova T, Solovchenko A, Baulina O, Lobakova E, Ivanova A, et al Versatility of the green microalga cell vacuole function as revealed by analytical transmission electron microscopy. Protoplasma. 2017;254: 1323–1340. 10.1007/s00709-016-1024-5 27677801 10.1007/s00709-016-1024-5

[28] Pun T. A new method for grey-level picture thresholding using the entropy of the histogram. Signal processing. 1980;2: 223–237.

[29] Pun T. Entropic thresholding, a new approach. Computer Graphics and Image Processing. 1981;16: 210–239.

[30] Sahoo PK, Soltani SA, Wong AK. A survey of thresholding techniques. Computer vision, graphics, and image processing. 1988;41: 233–260.

[31] Pal NR, Pal SK. Entropy: A new definition and its applications. IEEE transactions on systems, man, and cybernetics. 1991;21: 1260–1270.

[32] Sahoo PK, Slaaf DW, Albert TA. Threshold selection using a minimal histogram entropy difference. Optical Engineering. 1997;36: 1976–1982.

[33] Kapur JN, Sahoo PK, Wong AK. A new method for gray-level picture thresholding using the entropy of the histogram. Computer vision, graphics, and image processing. 1985;29: 273–285.

[34] Chang CI, Chen K, Wang J, Althouse ML. A relative entropy-based approach to image thresholding. Pattern recognition. 1994;27: 1275–1289.

[35] Lee SS, Horng SJ, Tsai HR. Entropy thresholding and its parallel algorithm on the reconfigurable array of processors with wider bus networks. IEEE Transactions on Image Processing. 1999;8: 1229–1242. 10.1109/83.784435 18267540 10.1109/83.784435

[36] Chang CI, Du Y, Wang J, Guo SM, Thouin PD. Survey and comparative analysis of entropy and relative entropy thresholding techniques. IEE Proceedings-Vision, Image and Signal Processing. 2006;153: 837–850.

[37] Trebbia P, Bonnet N. EELS elemental mapping with unconventional methods I. Theoretical basis: Image analysis with multivariate statistics and entropy concepts. Ultramicroscopy. 1990;34: 165–178. 2288036 10.1016/0304-3991(90)90070-3

[38] Trebbia P, Mory C. EELS elemental mapping with unconventional methods II. Applications to biological specimens. Ultramicroscopy. 1990;34: 179–203. 2288037 10.1016/0304-3991(90)90071-s

[39] Gorelova OA, Baulina OI, Solovchenko AE, Chekanov KA, Chivkunova OB, Fedorenko TA, et al Similarity and diversity of the *Desmodesmus* spp. microalgae isolated from associations with White Sea invertebrates. Protoplasma. 2015;252: 489–503. 10.1007/s00709-014-0694-0 25189657 10.1007/s00709-014-0694-0

[40] Rippka R, Deruelles J, Waterbury JB, Herdman M, Stanier RY. Generic assignments, strain histories and properties of pure cultures of cyanobacteria. Microbiology. 1979;111: 1–61.

[41] Moudříková S, Sadowsky A, Metzger S, Nedbal L, Mettler-Altmann T, Mojzeš P. Quantification of polyphosphate in microalgae by Raman microscopy and by a reference enzymatic assay. Analytical Chemistry. 2017;89: 12006–12013. 10.1021/acs.analchem.7b02393 29099580 10.1021/acs.analchem.7b02393

[42] Sicko-Goad L. A morphometric analysis of algal response to low dose, short-term heavy metal exposure. Protoplasma. 1982;110: 75–86.

[43] Sicko-Goad L, Lazinsky D. Quantitative ultrastructural changes associated with lead-coupled luxury phosphate uptake and polyphosphate utilization. Archives of Environmental Contamination and Toxicology. 1986;15: 617–627.

[44] Lazinsky D, Sicko-Goad L. Morphometric analysis of phosphate and chromium interactions in *Cyclotella meneghiniana*. Aquatic toxicology. 1990;16: 127–139.

[45] Korzhenevskaya TG, Gorelova OA, Baulina OI, Gusev MV. Accumulation of reserve polymers by *Nostoc muscorum* CALU 304 cells grown in mixed culture with plant tissue. Microbiology. 1999;68: 158–163.

[46] Gorelova OA, Korzhenevskaya TG. Formation of giant and ultramicroscopic forms of *Nostoc muscorum* CALU 304 during cocultivation with *Rauwolfia* tissues. Microbiology. 2002;71: 563–569. 12449632

[47] Gorelova OA, Kleimenov SY. The accumulation and degradation dynamics of cyanophycin in cyanobacterial cells grown in symbiotic associations with plant tissues and cells. Microbiology. 2003;72: 318–326. 12901011

[48] Lawry NH, Simon RD. The normal and induced occurrence of cyanophycin inclusion bodies in several blue-green algae. Journal of Phycology. 1982;18: 391–399.

[49] Pun T, Ellis JR, Eden M. Weighted least squares estimation of background in EELS imaging. Journal of microscopy. 1985;137: 93–100. 3973919 10.1111/j.1365-2818.1985.tb02565.x

[50] Hupfer M, Glöss S, Schmieder P, Grossart HP. Methods for detection and quantification of polyphosphate and polyphosphate accumulating microorganisms in aquatic sediments. International Review of Hydrobiology. 2008;93: 1–30.

[51] Majed N, Li Y, Gu AZ. Advances in techniques for phosphorus analysis in biological sources. Current opinion in biotechnology. 2012;23: 852–859. 10.1016/j.copbio.2012.06.002 22795053 10.1016/j.copbio.2012.06.002

[52] Heintzmann R, Ficz G. Breaking the resolution limit in light microscopy. Briefings in Functional Genomics. 2006;5: 289–301. 10.1093/bfgp/ell036 17170013

[53] Simon RD. Measurement of the cyanophycin granule polypeptide contained in the blue-green alga *Anabaena cylindrica*. Journal of bacteriology. 1973;114: 1213–1216. 4197270 10.1128/jb.114.3.1213-1216.1973 PMC285384

[54] Erickson NA, Kolodny NH, Allen MM. A rapid and sensitive method for the analysis of cyanophycin. Biochimica et Biophysica Acta (BBA)-General Subjects. 2001;1526: 5–9. 11287115 10.1016/s0304-4165(01)00098-8

[55] Lorenz B, Schröder HC. Methods for investigation of inorganic polyphosphates and polyphosphate-metabolizing enzymes In: Schröder HC, Müller WEG, editors. Inorganic polyphosphates. Progress in molecular and subcellular biology, vol 23 Springer, Berlin, Heidelberg; 1999 pp. 217–239. 10.1007/978-3-642-58444-2_11 10448679

[56] Ohtomo R, Sekiguchi Y, Kojima T, Saito M. Different chain length specificity among three polyphosphate quantification methods. Analytical biochemistry. 2008;383: 210–206. 10.1016/j.ab.2008.08.002 18727912 10.1016/j.ab.2008.08.002

[57] Kulakova AN, Hobbs D, Smithen M, Pavlov E, Gilbert JA, Quinn JP, et al Direct quantification of inorganic polyphosphate in microbial cells using 4′-6-diamidino-2-phenylindole (DAPI). Environmental science & technology. 2011;45: 7799–7803. 21875055 10.1021/es201123r

