# Supplementary figures and images for: A new simple method for quantification and locating P and N reserves in microalgal cells based on energy-filtered transmission electron microscopy (EFTEM) elemental maps
Source: PLoS One. 2018 Dec 11;13(12):e0208830. doi: 10.1371/journal.pone.0208830 (PMC6289464; doi:10.1371/journal.pone.0208830)

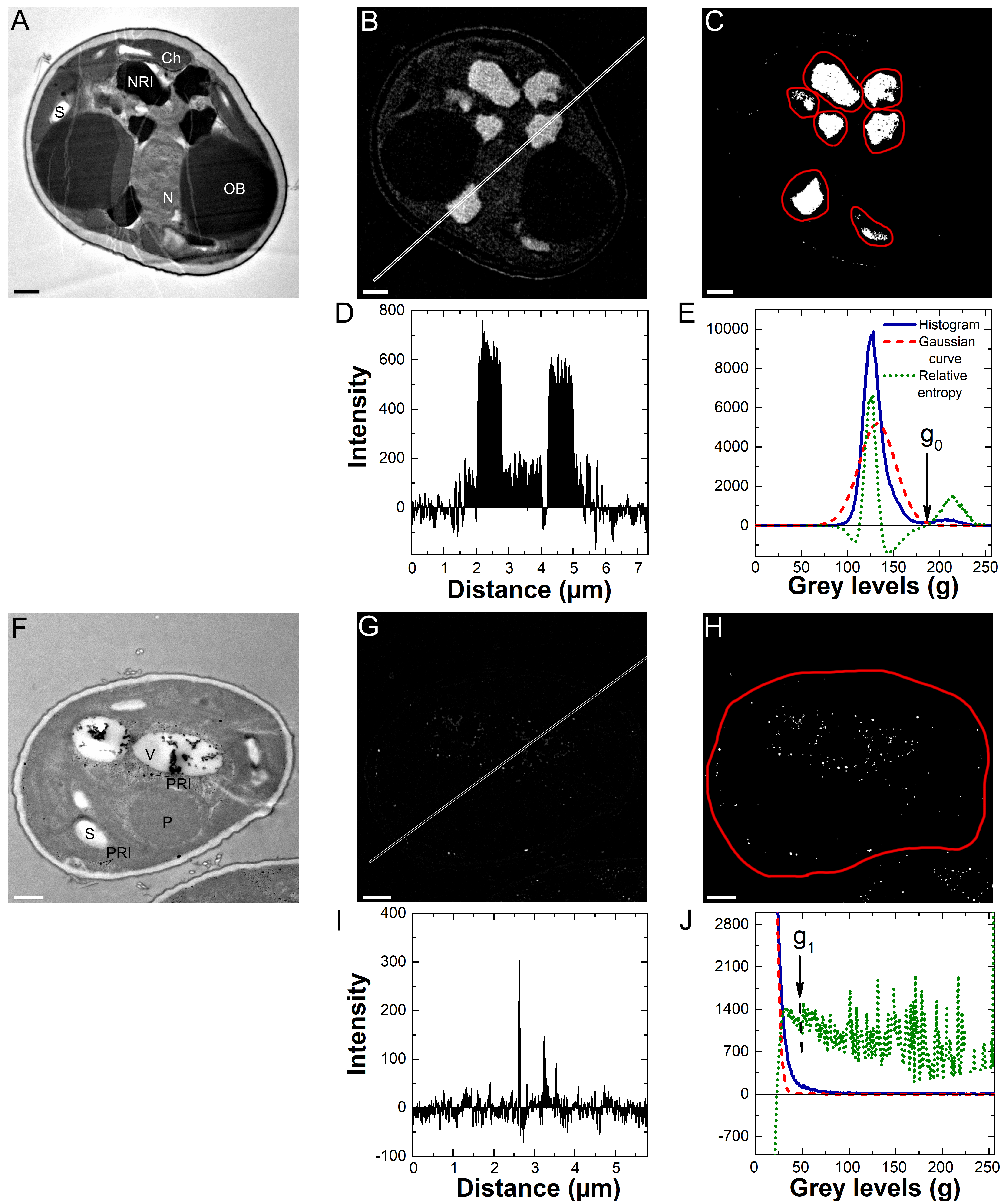

Supplement: S2 Fig — (A-E) Application to N-map of cell from P-starved culture. (F-J) Application to P-map of cell from P-sufficient stationary phase culture. (A) and (F) Elastically filtered TEM images of cell sections. (B) and (G) EFTEM maps of the cell sections. (D) and (I) Averaged profiles of the EFTEM maps. (C) and (H) The EFTEM maps processed according to the workflow “A” and “B”, respectively (see text and Fig 1). (E) and (J) The relative entropy analysis of the EFTEM maps (B) and (G), respectively. The averaged profiles were recorded along the white lines (see the maps). The red outline on the processed maps (C) and (H) indicates the region taken for the inclusion area measurements. In the graphs (E) and (J) the threshold pixels g0 and g1, respectively, used for the EFTEM maps processing are designated (for details see text). Ch chloroplast, N nucleus, NRI nitrogen-rich inclusion, OB oil body, P pyrenoid, PRI P-rich inclusion, S starch, V vacuole. Scale bars = 0.5 μm. (TIF) [file pone.0208830.s004.tif]

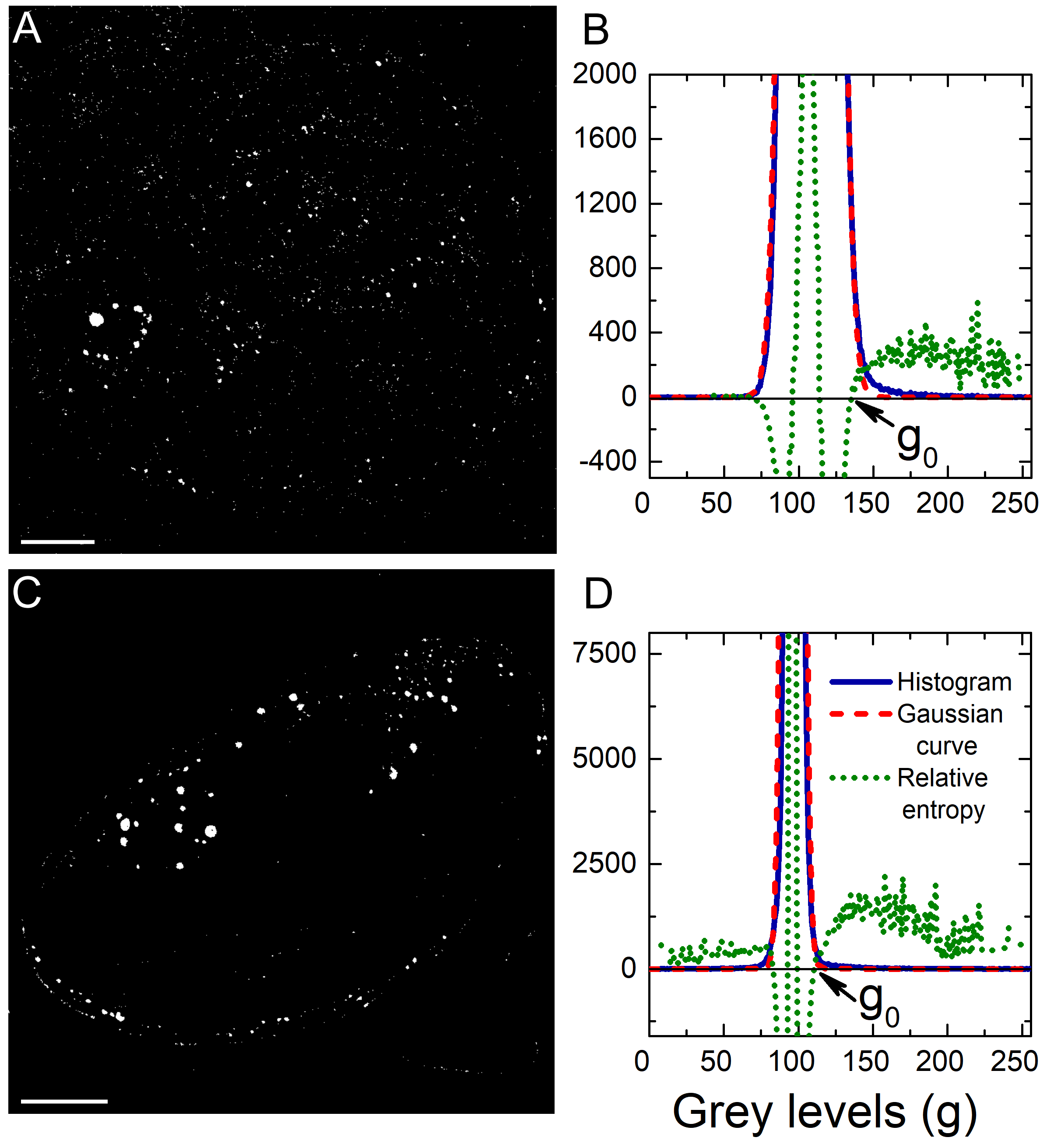

Supplement: S4 Fig — (A) and (B) The results of the workflow application to a cyanobacterium Nostoc sp. PCC 7118 from the P-sufficient stationary phase culture. (C) and (D) The results of the workflow application to a eukaryotic microalga Chlorella vulgaris IPPAS C-1 from the P-sufficient stationary phase culture. (A) and (C) The EFTEM maps processed according to the workflow “A” (see text and Fig 1). (B) and (D) The relative entropy analysis via the workflow “A” of the EFTEM P-maps from Fig 4B and 4G, respectively. In the graphs (B) and (D) the threshold pixels g0 used for the EFTEM maps processing are designated (for details see text). The P-rich inclusions on the processed maps (A) and (C) are surrounded by the pixels belonging to other structures. Scale bars = 0.5 μm. (TIF) [file pone.0208830.s006.tif]
